# Group A streptococcus isolated in Guyana with reduced susceptibility to β-lactam antibiotics

**DOI:** 10.1099/acmi.0.000746.v3

**Published:** 2024-06-20

**Authors:** Melissa Kalladeen, Paul Cheddie, Patrick Eberechi Akpaka

**Affiliations:** 1 Department of Paraclinical Sciences, University of the West Indies, St Augustine, Trinidad and Tobago; 2 Department of Medical Laboratory Science, University of Guyana, Turkeyen, Guyana

**Keywords:** antibiotic resistance, group A streptococci, Guyana, minimum inhibitory concentration, penicillin-binding proteins, *Streptococcus pyogenes*

## Abstract

**Introduction.***Streptococcus pyogenes* [group A streptococci (GAS)] is the causative agent of pharyngitis and various other syndromes involving cellulitis, streptococcal toxic shock syndrome (STSS), and necrotising fasciitis. Although the prevalence of GAS infections globally remains high, necessitating the widespread use of β-lactam antibiotics, GAS have remained largely susceptible to these agents. However, there have been several reports of GAS with reduced susceptibility harbouring mutations in genes for penicillin-binding proteins (PBPs). The objectives of this study were to examine the *in vitro* β-lactam susceptibility patterns of group A streptococci, determine the prevalence of drug resistance, and ascertain whether such resistance could be attributed to mutations in specific PBP genes.

**Methods.** In this study, we sought to use Sanger sequencing to identify mutations in PBP genes of *Streptococcus pyogenes* isolated from patients that required inpatient and outpatient care that could confer reduced PBP affinity for penicillin and/or cephalosporin antibiotics. All isolates were screened for susceptibility to penicillin, amoxicillin, and cefazolin using E-test strips.

**Results.** While there were no documented cases of reduced susceptibility to penicillin or amoxicillin, 13 isolates had reduced susceptibility to cefazolin. Examination of *pbp1a* by Sanger sequencing revealed several isolates with single amino acid substitutions, which could potentially reduce the affinity of PBP 1A for cefazolin and possibly other first-generation cephalosporins.

**Conclusion.** Penicillin and penicillin-derived antibiotics remain effective treatment options for GAS infections, but active surveillance is needed to monitor for changes to susceptibility patterns against these and other antibiotics and understand the genetic mechanisms contributing to them.

## Data Summary

The authors confirm all supporting data, code and protocols have been provided within the article or through supplementary data files. The genomic sequence data that support the findings of this study are openly available in GenBank (OR876961.1, OR876962.1, OR876963.1, OR876964.1, OR876965.1, OR876966.1, OR876967.1).

## Introduction

*Streptococcus pyogenes* [group A streptococcus (GAS)] is the causative agent of numerous diseases, such as streptococcal toxic shock syndrome (STSS) and necrotising fasciitis. Some of these cases may involve multiple syndromes [1]. Invasive group A streptococcal infections are estimated to be responsible for more than 500  000 deaths annually [2,3].

While beta-lactam antibiotics have remained effective therapeutic options for *S. pyogenes*, there have been reports of reduced susceptibility to these antibiotics [3]. There have been several explanations offered for this phenomenon, which include: (a) the poor penetration of penicillin in some tissue types (e.g. tonsillar epithelial cells), which leads to intracellular *S. pyogenes* persistence [4,5]; (b) the protection offered by β-lactamase-producing bacteria that are commonly present in the same infection sites as * S. pyogenes* (e.g. *Staphylococcus aureus*, *Moraxella catarrhalis*, *Haemophilus* spp.) [4,6, 7]; (c) the fact that there can be coaggregation between *M. catarrhalis* and *S. pyogenes*, which improves *S. pyogenes* adherence and colonization to human epithelial cells [6]; and (d) the presence of mutations in penicillin-binding proteins (PBPs) that under selective pressure can lead to the reduced susceptibility to β-lactams [8,9]. Although the number of reports of *S. pyogenes* with reduced susceptibility to β-lactams remains lower than other streptococci (e.g. *Streptococcus pneumoniae* and viridans group streptococci), tracking the emergence of such strains requires continuous surveillance.

To the best of the authors’ knowledge, there have been no confirmed reports of *S. pyogenes* beta-lactam resistance in Guyana. However, the antimicrobial surveillance system in the country is severely lacking and there are few mechanisms in place to determine the epidemiology of invasive *S. pyogenes* infection or to effectively document the susceptibility profiles of clinical isolates countrywide, and there is no system in place for investigating the genetic mechanisms responsible for reduced drug susceptibility. Hence, this study sought to determine the β-lactam susceptibility patterns of *S. pyogenes* isolated from patients receiving inpatient and outpatient care in Georgetown, Guyana, and to determine whether any observable resistance or reduced susceptibility might be attributed to mutations in genes encoding penicillin-binding protein.

## Methods

### Study location, time frame, and method of sampling

The *S. pygoenes* isolates used in this study were obtained from several sites: (a) a 600-bed tertiary care public hospital, identified as site ‘A’; (b) two private general medical and surgical hospitals, identified as sites ‘B’ and ‘C’; (c) from a fourth site, identified as site ‘D’, which is a multi-location private laboratory that provides testing and ambulatory care for a wide range of medical conditions. Although each of these institutions is in Georgetown, several of their patients come from the different geographical regions of Guyana.

Isolates from GAS infections were recovered at the various institutions from June 2021 to July 2022. In most instances the laboratories at each institution only employed a single method for identifying *S. pyogenes* (e.g. bacitracin susceptibility or PYR test). For this reason, all beta-haemolytic streptococci that were presumptively identified as *S. pyogenes* were included in this study.

### Culture, identification, and antibiotic susceptibility testing of GAS

**Fig. 1.**
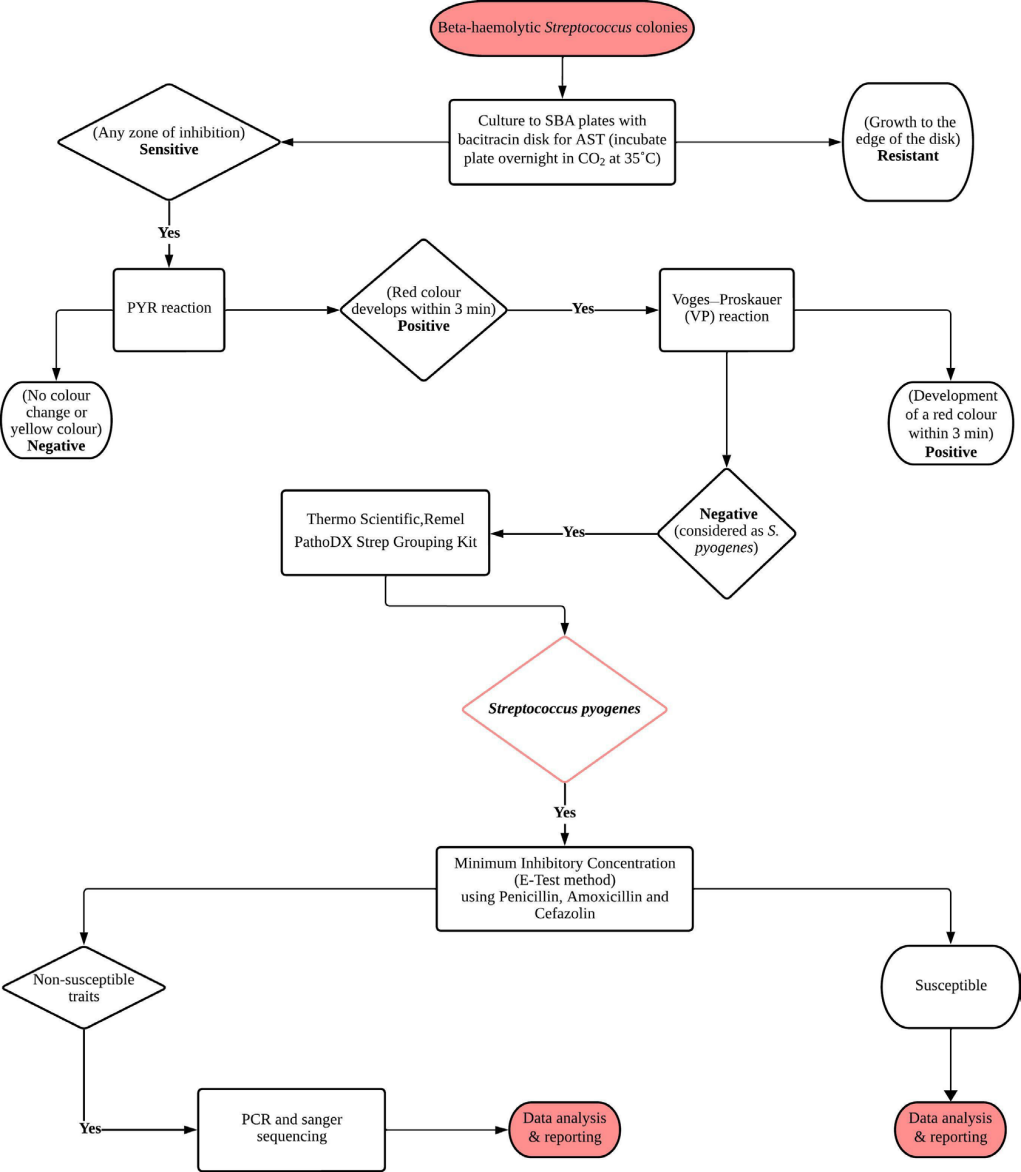
Algorithm for identifying *S. pyogenes*.

GAS received from the various institutions were subjected to additional testing based on the following protocol (Fig. 1): ‘Conventional methods for isolation and Identification of Streptococcus pyogenes’ (http://dx.doi.org/10.17504/protocols.io.rm7vzx6e2gx1/v1).

Antibiotic susceptibility testing of laboratory-confirmed *S. pyogenes* isolates was conducted using the E-test method with penicillin, cefazolin, and amoxicillin minimum inhibitory concentration (MIC) strips. Briefly, colonies of *S. pyogenes* grown overnight were used to prepare a suspension in Mueller–Hinton broth equivalent to a 0.5 McFarland standard (~1 x 10^8^ c.f.u. ml^−1^). A sterile swab was dipped into the broth and used to streak the surface of a culture plate (90×15 mm) containing Mueller–Hinton (MH) agar with 5 % sheep blood. Using aseptic techniques, the MIC strips were gently pressed onto the surface of the agar. The plates were incubated in an inverted position at 35–37 °C in 5 % CO_2_ atmosphere for 24 h. Quality control for this phase of testing was performed using *S. pyogenes* ATCC 19615. The minimum inhibitory concentration was identified as the point where the edge of the inhibition ellipse intersected the strip. The MIC results were interpreted per the CLSI M100-S31 guidelines [10].

### DNA extraction and Sanger sequencing

*S. pyogenes* isolates with reduced β-lactam susceptibility were processed using a crude DNA extraction protocol to prepare them for sequencing. Briefly, five to seven colonies of overnight growth of *S. pyogenes* were suspended in 1 ml of molecular-grade water in a DNase/RNase-free micro-centrifuge tube. The micro-centrifuge tube was then boiled at 100 °C for 10 min in a water bath before being centrifuged for 5 min at 1 000 r.p.m. The supernatant was removed and placed into a DNase/RNase-free cryogenic vial and stored at −20 °C. The frozen sample was then shipped to GENEWIZ (RRID:SCR_003177) for further analysis using Sanger sequencing. Using their proprietary methodology, they performed polymerase chain reaction (PCR) on purified genomic DNA, amplifying an internal region <1 kb. Using a template, they sequenced a target region of the PCR transcript. For the identification of substitutions and deletions in the amino acid sequence of *pbp1a* and *pbp2x*, NCTC12064 (GenBank accession no. NZ_LS483338.1) was used as the *S. pyogenes* reference strain.

### Data analysis

The MIC_50_ (i.e., MIC at which 50 % of isolates were inhibited) and MIC_90_ (i.e. MIC at which 90 % of isolates were inhibited) were calculated based on the susceptibility test results. The CLSI guidelines do not provide clinical breakpoints for cefazolin and, as such, an assessment of the MIC distribution for this agent was performed using an epidemiological cut-off value (ECV) as described by other authors [11]. Briefly, the 24 h MIC distributions for cefazolin from this study were collected and used to determine the modal MIC. The MIC distributions were then aggregated and used to establish the wild-type MIC distributions and ECVs. To the best of our knowledge, this was the first study to investigate infections caused by GAS and, currently, in Guyana, there is no national database for collecting MIC data for GAS. Therefore, we assumed that the ECV would be the MIC that encompassed ≥95 % of all MIC values in the wild-type MIC distributions [11].

Sequencing results were analysed using NCBI blast and sequence alignment for the detection of substitutions and deletions was performed using SnapGene v. 7.0.2 (RRID:SCR_015052). The Kyoto Encyclopedia of Genes and Genomes (KEGG) GENES database (https://www.genome.jp/kegg/genes.html) was used to define the transglycosylase and transpeptidase domains of the PBPs. SPy_1649 was used as a reference for PBP 1A. Sequences with amino acid substitutions or deletions along the PBP gene were considered to be possible candidates for lower β-lactam affinity and contributing to increased MICs for these antibiotics.

## Results

### Bacterial isolates

Fifty-three beta-haemolytic isolates were presumptively identified as *S. pyogenes* from three of the four institutions that participated in this study. Most of these isolates were obtained from site A (*n*=42). The other institutions contributed 11 isolates (9 from site D, 2 from site B, and none from site C). Of the 53 isolates, only 37.7 % (*n*=20) were confirmed as *S. pyogenes* using conventional identification methods.

### Antimicrobial susceptibility testing

All 20 laboratory-confirmed *S. pyogenes* isolates were subjected to antibiotic susceptibility testing using the E-test method with penicillin, cefazolin, and amoxicillin. All isolates were susceptible to penicillin and amoxicillin (Table 1).

**Table 1.** Source and β-lactam susceptibility profile for laboratory-confirmed GAS

Clinical isolate	Source facility	Specimen type	Antibiotic agents (MIC, mg ml^−1^)		
			Penicillin S≤**0.12**	Cefazolin	Amoxicillin S≤**0.12**
S-001	A	Pharyngeal swab	0.016	0.125	0.023
S-002	A	Urine	0.023	0.19	0.064
S-003	A	Wound	0.023	0.125	0.016
S-004	A	Urine	0.016	0.19	0.016
S-006	A	Pharyngeal swab	0.016	0.38	0.016
S-010	A	Pharyngeal swab	0.016	0.19	0.016
S-017	A	Pharyngeal swab	0.016	0.19	0.023
S-023	A	Pharyngeal swab	0.023	0.19	0.016
S-026	A	Pharyngeal swab	0.016	0.125	0.023
S-027	A	Wound	0.016	0.19	0.023
S-030	A	Pharyngeal swab	0.016	0.19	0.023
S-035	D	Blood	0.016	0.25	0.023
S-036	D	Wound	0.023	0.75	0.016
S-037	D	Urine	0.023	0.125	0.023
S-046	A	Pharyngeal swab	0.016	0.125	0.023
S-047	A	Throat swab	0.016	0.25	0.016
S-048	A	Sputum	0.032	0.125	0.016
S-049	D	Sputum	0.016	0.19	0.023
S-050	D	Sputum	0.023	0.25	0.023
S-051	D	Sputum	0.016	0.125	0.023

The MIC for penicillin ranged from 0.016 to 0.032 µg ml^−1^ for all the strains (*n*=20). The MIC_50_ and MIC_90_ were 0.016 and 0.023 µg ml^−1^, respectively. For amoxicillin, the MICs observed ranged from 0.016 to 0.064 µg ml ^−1^. The MIC_50_ and MIC_90_ values were both 0.023 µg ml.

For cefazolin, there were seven isolates that had MICs of 0.12 µg ml^−1^. Thirteen isolates had higher MICs, ranging from 0.19 to 0.75 µg ml^−1^. As there was no clinical breakpoint for cefazolin for *S. pyogenes*, we investigated the wild-type MIC distribution of cefazolin using the MIC of the reference organism, i.e. *S. pyogenes* ATCC 19615 (MIC=0.09), and all the isolates tested. This was done because of the absence of published or reported MICs for group A streptococci locally. Using a 95 % epidemiological cut-off value (ECV) [11], the wild-type ECV was determined to be 0.19. All 13 isolates had MICs ≥0.19 and were considered to have reduced susceptibility to cefazolin when compared to the wild-type strains. Eight of these isolates were recovered from patients hospitalised at site A, while five were from site D.

### PCR and Sanger sequencing

Based on the results from susceptibility testing, the 13 isolates adjudged to have reduced cefazolin susceptibility were further assessed to understand whether the reduced drug susceptibility was linked to genetic modifications in their penicillin-binding proteins. These isolates were subjected to crude DNA extraction, and the extracted DNA was sent to GENEWIZ (RRID:SCR_003177) for DNA purification, PCR, and Sanger sequencing of the PBP 1A and PBP 2X genes.

The results revealed that there were substitutions in the amino acid sequence of the transglycosylase-transpeptidase region of *pbp1a* (Table 2). These substitutions were considered as possible causes of the reduced PBP affinity for β-lactams.

**Table 2.** Deduced amino acid substitutions in the PBP1a gene of the 13 isolates identified as *Streptococcus pyogenes*

Clinical isolate	Position of mutation in *pbp1a*
S-002	–
S-004	–
S-006	H291N, I337V, I361T
S-010	–
S-017	–
S-023	–
S-027	I361T
S-030	T556A
S-035	H291N, I337V, I361T
S-036	H291N, I337V, I361T
S-047	–
S-049	H291N, I337V, I361T
S-050	I486M, I645M

Unfortunately, sequencing of *pbp2x* could not be performed because degradation of the primary genomic sequences prevented reliable PCR and Sanger sequencing.

## Discussion

This study describes, to the best of our knowledge, the first reported cases of group A streptococci that exhibited reduced susceptibility to a β-lactam antibiotic. Based on the results obtained, it is evident that penicillin and amoxicillin were the most effective treatment options for *S. pyogenes* infections. These results were not entirely unexpected, as they were similar to those of Camara *et al*. [12] and several other studies performed in other parts of the world, including Morocco [13], France [14], and Germany [15]. However, of the 20 laboratory-confirmed *S. pyogenes* isolates identified, 13 were deemed to express reduced susceptibility to cefazolin when compared to the wild-type strain. This finding was somewhat unusual, given that cephalosporins have been described as being superior to penicillin antibiotics for GAS infections [16]. Cefazolin is recommended for treating respiratory infections with *S. pyogenes* in adults and paediatric patients [17]. First-generation cephalosporins, like cefazolin and cephalexin, also represent effective first-line therapeutic alternatives to penicillin for GAS infections in patients at risk of anaphylaxis due to penicillin [18,19]. Although the CLSI interpretive guideline does not provide a clinical breakpoint for cefazolin and, given that this was the first research to examine cefazolin susceptibility among GAS in Guyana, an epidemiological cut-off that encompasses 95 % of the wild-type strains was considered. Therefore, we assumed that all isolates that had MICs below the ECV were ‘susceptible’ and those with MICs at or above the cut-off were assumed to have reduced susceptibility relative to their wild-type counterparts. This approach was based on work done by several other authors [11,20, 21].

Among the proposed mechanisms contributing to reduced susceptibility of streptococci to cephalosporins and other β-lactam antibiotics, the role of penicillin-binding proteins (PBPs) has received much attention. In particular, the alteration of PBPs resulting in their low affinity for these antibiotics or the acquisition of new PBPs have been identified as key resistance determinants in *S. pneumoniae* and viridans streptococci [22,25]. While research has shown that cephalosporin resistance is linked to alterations to PBPs 1A and 2X [26], mutations of these PBPs in *S. pyogenes* are not widespread [27], although there have been reports that identified a single amino acid mutation of *pbp2x* as a mediator of increased MICs for β-lactam in *S. pyogenes* [28,29]. Furthermore, it has been shown that alterations in PBP1A can help to potentiate low- and high-level β-lactam resistance in other streptococcal species [30,31].

To investigate the role of mutations in *pbp1a* and *pbp2x* in mediating reduced susceptibility to cefazolin, the 13 isolates were subjected to PCR and Sanger sequencing. We found that seven of the isolates (GenBank accession nos OR876961.1, OR876962.1, OR876963.1, OR876964.1, OR876965.1, OR876966.1, OR876967.1) had amino acid substitutions near to and within the conserved active motif of *pbp1a*, with four of these isolates having the same amino acid substitutions (i.e. histidine for asparagine at position 291, isoleucine for valine at position 337, and isoleucine for threonine at position 361). It is conceivable that these amino acid substitutions caused the reduced affinity of the PBP for cefazolin but not penicillin. This is not unusual, given that point mutations that affect the affinity of a PBP for one β-lactam antibiotic may have little or no effect for another [28]. Furthermore, reduced susceptibility to cephalosporins is more commonly associated with alterations in both *pbp1a* and *pbp2x* [26]. Unfortunately, we were unable to sequence *pbp2x*.

Interestingly, the similarities between the amino acid substitutions in the four isolates suggests that these isolates could be closely related, thus representing a prevalent clone. However, further investigation is required to better understand and explain these findings. It seems entirely possible that repeated and poorly regulated antibiotic use may be essential factors driving reduced cephalosporin susceptibility. These factors are likely to select for GAS that have progressively accumulated amino acids substitutions in PBPs [9,22].

This is the first study, as far as the available literature suggests, to identify mutations in the *pbp1a* in GAS isolated from patients receiving inpatient and outpatient care in Guyana that likely caused reduced susceptibility to a first-generation β-lactam cephalosporin antibiotic in the country. These findings reinforce the importance of conducting active monitoring of β-lactam susceptibility among these isolates and utilising tools such as molecular testing to better understand the epidemiology of drug resistance and to study other genetic mechanisms contributing to it.

## Abbreviations

ATCC: American Type Culture Collection
CLSI: Clinical and Laboratory Standards Institute
ECV: epidemiological cut-off value
GAS: group A *Streptococcus*
KEGG: Kyoto Encyclopedia of Genes and Genomes
MIC: minimum inhibitory concentration
PBP: penicillin-binding protein

## References

[1] Lepoutre A, Doloy A, Bidet P, Leblond A, Perrocheau A (2011). Epidemiology of invasive *Streptococcus* pyogenes infections in France in 2007. J Clin Microbiol.

[2] World Health Organization (2023). Increased incidence of scarlet fever and invasive Group A Streptococcus infection - multi-country. Internet.

[3] Carapetis JR, Steer AC, Mulholland EK, Weber M (2005). The global burden of group A streptococcal diseases. Lancet Infect Dis.

[4] Cattoir V, Ferretti JJ, Stevens DL, Fischetti VA (2022). Streptococus Pyogenes: Basic Biology to Clinical Manifestations.

[5] Kaplan EL, Chhatwal GS, Rohde M (2006). Reduced ability of penicillin to eradicate ingested group A streptococci from epithelial cells: clinical and pathogenetic implications. Clin Infect Dis.

[6] Brook I (2013). Penicillin failure in the treatment of streptococcal pharyngo-tonsillitis. Curr Infect Dis Rep.

[7] Schaar V, Uddbäck I, Nordström T, Riesbeck K (2014). Group A streptococci are protected from amoxicillin-mediated killing by vesicles containing β-lactamase derived from Haemophilus influenzae. J Antimicrob Chemother.

[8] Pichichero ME, Casey JR (2007). Systematic review of factors contributing to penicillin treatment failure in *Streptococcus* pyogenes pharyngitis. Otolaryngol Head Neck Surg.

[9] Yu D, Guo D, Zheng Y, Yang Y (2023). A review of penicillin binding protein and group A *Streptococcus* with reduced-β-lactam susceptibility. Front Cell Infect Microbiol.

[10] Clinical and Laboratory Standards Institute (2021). The Clinical and Laboratory Standards Institute.

[11] Pfaller MA, Espinel-Ingroff A, Canton E, Castanheira M, Cuenca-Estrella M (2012). Wild-type MIC distributions and epidemiological cutoff values for amphotericin B, flucytosine, and itraconazole and *Candida* spp. as determined by CLSI broth microdilution. J Clin Microbiol.

[12] Camara M, Dieng A, Boye CSB (2013). Antibiotic susceptibility of streptococcus pyogenes isolated from respiratory tract infections in Dakar, Senegal. Microbiol Insights.

[13] Benouda A, Sibile S, Ziane Y, Elouennass M, Dahani K (2009). Place de *Streptococcus* pyogenes dans les angines au Maroc et état actuel de sa sensibilité aux antibiotiques. Pathologie Biologie.

[14] Mariani-Kurkdjian P, Doit C, Deforche D, Brahimi N, Francois M (2004). Sensibilité actuelle en France de *Streptococcus* pyogenes responsable d’angine aiguë. La Presse Médicale.

[15] Sauermann R, Gattringer R, Graninger W, Buxbaum A, Georgopoulos A (2003). Phenotypes of macrolide resistance of group A streptococci isolated from outpatients in Bavaria and susceptibility to 16 antibiotics. J Antimicrob Chemother.

[16] Casey JR, Pichichero ME (2007). The evidence base for cephalosporin superiority over penicillin in streptococcal pharyngitis. Diagn Microbiol Infect Dis.

[17] US Food and Drug Administration (2023). Cefazolin for Injection, USP. Highlights of Prescribing Information.

[18] Sousa-Pinto B, Blumenthal KG, Courtney L, Mancini CM, Jeffres MN (2021). Assessment of the frequency of dual allergy to penicillins and cefazolin: a systematic review and meta-analysis. JAMA Surg.

[19] Chaudhry SB, Veve MP, Wagner JL (2019). Cephalosporins: a focus on side chains and β-lactam cross-reactivity. Pharmacy.

[20] Dalhoff A, Ambrose PG, Mouton JW (2009). A long journey from minimum inhibitory concentration testing to clinically predictive breakpoints: deterministic and probabilistic approaches in deriving breakpoints. Infection.

[21] Badr MT, Blümel B, Baumgartner S, Komp JMA, Häcker G (2020). Antimicrobial susceptibility patterns and wild-type MIC distributions of anaerobic bacteria at a German University Hospital: a five-year retrospective study (2015-2019). Antibiotics.

[22] Hakenbeck R, Coyette J (1998). Resistant penicillin-binding proteins. Cell Mol Life Sci.

[23] Zapun A, Contreras-Martel C, Vernet T (2008). Penicillin-binding proteins and beta-lactam resistance. FEMS Microbiol Rev.

[24] Engel H, Mika M, Denapaite D, Hakenbeck R, Mühlemann K (2014). A low-affinity penicillin-binding protein 2x variant is required for heteroresistance in *Streptococcus pneumoniae*. Antimicrob Agents Chemother.

[25] Amoroso A, Demares D, Mollerach M, Gutkind G, Coyette J (2001). All detectable high-molecular-mass penicillin-binding proteins are modified in a high-level beta-lactam-resistant clinical isolate of *Streptococcus mitis*. Antimicrob Agents Chemother.

[26] Dowson CG, Barcus V, King S, Pickerill P, Whatmore A (1997). Horizontal gene transfer and the evolution of resistance and virulence determinants in *Streptococcus*. J Appl Microbiol.

[27] Hayes A, Lacey JA, Morris JM, Davies MR, Tong SYC (2020). Restricted sequence variation in *Streptococcus* pyogenes penicillin binding proteins. mSphere.

[28] Vannice KS, Ricaldi J, Nanduri S, Fang FC, Lynch JB (2020). *Streptococcus* pyogenes pbp2x mutation confers reduced susceptibility to β-lactam antibiotics. Clin Infect Dis.

[29] Olsen RJ, Zhu L, Musser JM (2020). A single amino acid replacement in penicillin-binding protein 2X in *Streptococcus* pyogenes significantly increases fitness on subtherapeutic benzylpenicillin treatment in a mouse model of necrotizing myositis. Am J Pathol.

[30] Haenni M, Moreillon P (2006). Mutations in penicillin-binding protein (PBP) genes and in non-PBP genes during selection of penicillin-resistant *Streptococcus gordonii*. Antimicrob Agents Chemother.

[31] Job V, Carapito R, Vernet T, Dessen A, Zapun A (2008). Common alterations in PBP1a from resistant *Streptococcus pneumoniae* decrease its reactivity toward beta-lactams: structural insights. J Biol Chem.

